# Understanding why cancer patients accept or turn down psycho-oncological support: a prospective observational study including patients’ and clinicians’ perspectives on communication about distress

**DOI:** 10.1186/s12885-017-3362-x

**Published:** 2017-05-30

**Authors:** Diana Zwahlen, Theresa Tondorf, Sacha Rothschild, Michael T. Koller, Christoph Rochlitz, Alexander Kiss

**Affiliations:** 1 Department of Psychosomatic Medicine, University Hospital Basel, Hebelstrasse 2, 4031 Basel, Switzerland; 2 Medical Oncology Department, University Hospital Basel, Petersgraben 4, 4031 Basel, Switzerland; 3 Institute for Clinical Epidemiology and Biostatistics, University Hospital Basel, Basel, Switzerland

## Abstract

**Background:**

International standards prioritize introducing routine emotional distress screening in cancer care to accurately identify patients who most need psycho-oncological treatment, and ensure that patients can access appropriate supportive care. However, only a moderate proportion of distressed patients accepts referrals to or uses psycho-oncological support services. Predictors and barriers to psycho-oncological support service utilization are under-studied. We know little about how patients and oncologists perceive the discussions when oncologists assess psychosocial distress with a screening instrument.

We aim to 1) assess the barriers and predictors of uptake of in-house psycho-oncological support along the distress screening pathway in cancer patients treated at a University Oncology Outpatient Clinic and, 2) determine how patients and clinicians perceive communication about psychosocial distress after screening with the Distress Thermometer.

**Methods:**

This is a quantitative prospective observational study with qualitative aspects. We will examine medical and demographic variables, cancer patient self-reports of various psychological measures, and aspects of the patient-clinician communication as variables that potentially predict uptake of psycho-oncological support service. We will also assess the patients’ reasons for accepting or refusing psycho-oncological support services. We assess at three points in time, based on paper-and-pencil questionnaires and two patient interviews during the study period. We will monitor outcomes (psycho-oncology service uptake) four months after study entry.

**Discussion:**

The study will improve our understanding of characteristics of patients who accept or refuse psycho-oncological support, and help us understand how patients’ and oncologists perceive communication about psychosocial distress, and referral to a psycho-oncologist. We believe this is the first study to focus on factors that affect uptake or rejection of psycho-oncological support services along the screening and referral pathway. The study 1) combines standard assessment with qualitative data collection, 2) embraces patient and oncologist perspectives, and, 3) focuses on patient-clinician communication about psychosocial issues raised by a standard screening instrument.

Our results may improve routine practices and eliminate barriers to adequate health care, and make it easier to recognize patients with high distress levels who underuse the service.

## Background

Routine distress screening identifies patients who experts believe most need psycho-oncological care. This screening is intended to give patients access to supportive care services. Distress screening also identifies comorbidities, including depression and anxiety. The Distress Thermometer (DT) is widely used and has been validated as a reliable and valid screening tool [1–5]. Distress screening is now an international standard in comprehensive care of cancer patients [6, 7] and, in many countries, a criterion for cancer center accreditation [8, 9]. In principle, distress screening should identify cancer patients in psychosocial distress and direct them to appropriate treatment services but, in practice, it is debatable whether they can solve the challenges posed by this process [10, 11].

Studies that investigated distress screening, referral, and acceptance of professional support service found low correspondence between emotional distress and uptake. Some studies found that patients who reported a higher burden of emotional symptoms were more likely to access services than those who reported a lower burden of symptoms [12–16]. Referral rates and resource utilization still seem low, given documented high levels of distress. Various studies report that distress correlates moderately or not at all with the wish for support or acceptance of a referral [17–19]. Distress scores and expert perspectives do not appear to reflect patients’ needs for psycho-oncological support. It would thus be very useful to be able to offer recommendations on managing discordance between patient preference and screening results.

In addition to the clinical screening and referral process, there are other potential predictors of psycho-oncological support service uptake than emotional distress. We have some evidence that patient characteristics are linked to psycho-oncological support uptake: being younger [15, 18, 20], being female [18, 21, 22], and being more highly educated [15, 21, 23, 24].

Gaining insight into the patient decision process for or against support uptake is difficult. Although the patient-clinician conversation is an important element in the screening and referral process [25, 26], we do not know how patients and physicians perceive communication after distress, or how their perceptions influence the referral process. Few studies investigate the subjective reasons people accept or reject psycho-oncological support services, and even fewer include qualitative components. A recent review [27] found the primary patient-reported reason for rejection is they perceive *no subjective need*. The second reason was lack of information about availability of psychological support services. Other patient explanations include a preference for self-managing symptoms, or the belief that help would be ineffective.

Our overarching aim is to assess factors along the distress screening and referral pathway, so we can map the process by which patients take decisions for or against uptake of psycho-oncological support service. In our prospective observational study, we will consider distress scores, medical and demographic variables, patient self-reports of psychological and social support measures, and aspects of the patient-clinician communication as potential predictors of uptake of psycho-oncological services. We also want to assess patient and physician perceptions of communication about psychosocial issues, spurred by a standard screening instrument. We will incorporate qualitative data and assess the reasons patients give us for accepting or refusing psycho-oncological support.

Our two principal research questions are: (1) Which factors along the screening pathway determine uptake of psycho-oncological support in ambulatory cancer patients; and, (2) When the DT stimulates conversations between patients and clinicians about psychosocial distress, how do they perceive those conversations?

## Methods

### Study design

This is a prospective, observational, quantitative single-center study in the Oncology Outpatient Clinic of the University Hospital Basel (Switzerland) medical center.

### Study participants

Cancer patients are consecutively recruited from the Oncology Outpatient Clinic, which receives approximately 600 new cancer patients per year. Patients are eligible to participate if they meet the following inclusion criteria: older than 18 years; diagnosed with any kind of solid tumor or hematologic malignancy; first consultation at the Oncology Outpatient Clinic; and, at least one more scheduled appointment. We exclude patients with insufficient command of the German language, and patients too physically weak or cognitively incapacitated to participate (evaluated by attending oncologists). Participating clinicians are oncologists and residents from the Medical Oncology Department.

### Standard screening and referral procedure

Routine distress screening and referral guidelines were implemented to conform to international guidelines [6] and are standard procedure in the Oncology Outpatient Clinic since 2012. Independent of study participation, patients are given a distress screening form (Distress Thermometer, DT) [6, 28] in the waiting area on the first visit at the Oncology Outpatient Clinic, shortly before their consultation. A nurse asks patients to fill in the questionnaire; patients then hand it to the attending oncologist. The back side of the questionnaire contains information about available professional psycho-oncological support services at the Oncology Outpatient Clinic. The oncologist discusses the score with the patient during the first consultation, if possible. Oncologists are advised to recommend psycho-oncological support to patients with clinically relevant level of distress, indicated by a score of 5 or higher on the DT. Mehnert et al. [28] guided by the patient’s DT score and their own estimation of the clinically relevant level of the patient’s distress, the oncologist recommends the patient to use psycho-oncological support services and discusses a referral. The patient’s wish guides the referral.

### Psycho-oncological support service at the Oncology Outpatient Clinic

The psycho-oncological team at the Oncology Outpatient Clinic is thoroughly integrated into the medical oncology team; it is situated on the ward and attends daily team meetings.

### Oncologists’ training on distress screening and communication

Oncologists were instructed about psycho-oncological procedures in a one-hour communication training that covered 1) how to discuss distress scores with patients, and, 2) how to refer patients to the psycho-oncological support service. An expert in the field of medical communication and co-investigator of the study conducted the training (A.K.).

### Study procedure

**Fig. 1:**
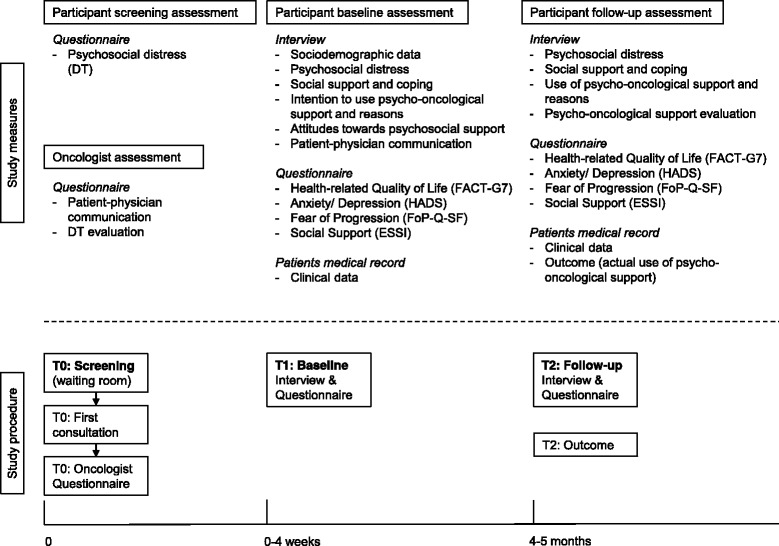
Overview of study procedure and study measures. Legend: DT: Distress Thermometer; HADS: Hospital Anxiety and Depression Scale; ESSI: ENRICHD social support inventory; FACT-G7: Health-related quality of life; FoP-Q-SF: Fear of Progression Questionnaire

Eligible patients are informed about the study by attending oncologists at their first consultation. All patients willing to participate are approached by the study coordinator after the first or second consultation on the ward, or contacted by telephone. Participants are then fully informed about the study in a separate room or by telephone and receive an informed consent form, a baseline questionnaire, and a return envelope (T0). After they provide written informed consent, participants are contacted for a baseline interview within four weeks after they are recruited (T1). Patient preference determines if the interview will be by telephone or face-to-face at the outpatient clinic. Four months later, participants receive a follow-up questionnaire by mail with a return envelope, and are contacted for a follow-up interview (T2). Oncologists complete a structured paper-and-pencil questionnaire after every first consultation with a new, eligible patient (T0). Figure 1 provides an overview of study procedure and study measures.

Summary of study visits:

- T0 (participant screening and oncologist assessment) takes place when patients fill out the DT form at their first consultation at the oncology outpatient clinic. Patients receive the distress screening form before the first consultation, and oncologists are instructed to fill out a questionnaire afterwards.
- T1 (participant baseline assessment) is the baseline assessment, a few days to four weeks after study recruitment. This assessment includes the baseline interview (telephone or face-to-face) and a paper-and-pencil questionnaire (filled out at home).
- T2 (participant follow-up assessment and outcome monitoring) takes place four months later by follow-up interview (telephone or face-to-face) and a paper-and-pencil questionnaire (filled out at home). Outcome is monitored at T2.

### Interview procedure and interviewer training

Interviews will be semi-structured and conversational. Interviewers will use prompts and reflections to encourage patients to talk, and will ask open and closed questions to elicit detail where necessary. Interviewers take notes on patients’ answers to open-ended questions. We will use content analysis to analyze patient answers (see section, *Analysis of qualitative data*). To guarantee the quality of the data collection process, we developed an interviewer’s manual that ensures interviews will be of equally high quality, regardless of interviewer. Interviewers will be trained and supervised by an experienced clinical psycho-oncologist (D.Z.).

### Ethics and data safety

This study will be conducted in accord with Declaration of Helsinki and Good Clinical Practice guidelines. The local Ethics Committee in Basel, Switzerland (EKNZ) approved the study (reference number EK 220/13). The EKNZ is eligible to approve studies in the University Hospital of Basel. Collected data will be de-identified and stored in a study-specific electronic database system, in a separate locker. A patient-specific identification number (Patient ID) is used to encode patient data. Patient identification data and patient study data will be stored separately. The translation key that links patient identification data to patient study data will be electronically and physically separate from the study database system.

### Sociodemographic data, clinical data, and oncologists’ personal data

Sociodemographic data (age, gender, relationship status, living status, children, education, profession, employment status, monthly household income) is collected during the baseline interview (T1). Clinical data (cancer type, tumor staging, stage of disease, weeks since diagnosis, treatment intention, current and past treatments, past cancer diagnoses, comorbidities, ECOG scale) is retrieved from the hospital’s electronic database and from patients’ medical records by an oncologist and co-author of the study (S.R.) at baseline (T1) and follow-up (T2). Oncologists’ personal data (age, gender, professional experience) is gathered by a single e-mail questionnaire.

### Primary outcome measure

The primary outcome is each participant’s attendance (yes/no) of the in-house psycho-oncological support service (at least one appointment) during the study period. Outcome data is retrieved from patients’ medical records and is ascertained by individual contact with the patient at T2.

### Secondary outcome measures

Secondary outcomes include: 1) agreement of and disparities between patient and clinician perceptions of communication about psychosocial distress and referral after DT screening (Table 1); 2) patients’ reasons for (non-)uptake of psycho-oncological support; 3) social support and coping measures; 4) psychosocial distress measures; and, 5) attitudes towards psycho-oncological support.

**Table 1:** Patients’ and oncologists’ perception of the first consultation

Variables	Patients’ Baseline Interview Questions (T1)	Oncologists’ Questionnaire Items (T0)
Talking about psychosocial distress	Q: “Did the oncologist talk about your psychosocial distress with you?”	Q: “Did you talk with the patient about his/her psychosocial distress?”
	A: Yes/ No/ Don’t remember	A: Yes/ No
Importance of talking about psychosocial distress	Q: “How important was it for you to talk about your psychosocial distress with the oncologist?”	Q: “How important was it for the patient to talk about his/her psychosocial distress?”
	A: Scale from 0 “not important at all” to 10 “very important”	A: scale from 0 “not important at all” to 10 “very important”
Information about psycho-oncological support	Q: “Did the oncologist inform you about the psycho-oncological support service?”	Q: “Did you inform the patient about the psycho-oncological support service?”
	A: Yes/ No/ Don’t remember	A: Yes/ No
Specification of psycho-oncological support	Q: “Did the oncologist inform you about how the psycho-oncologist can provide support?”	Q: “Did you inform the patient about how the psycho-oncologist can provide support?”
	A: Yes/ No/ Don’t remember	A: Yes/ No
Recommendation of psycho-oncological support	Q: “Did the oncologist recommend that you attend the psycho-oncological support service?”	Q: “Did you recommend that the patient attends the psycho-oncological support service?”
	A: Yes/ No/ Don’t remember	A: Yes/ No
Helpfulness of psycho-oncological support	Q: “How helpful do you think psycho-oncological support would be for yourself?”	Q: “How helpful do you think psycho-oncological support would be for the patient?”
	A: scale from 0 “not helpful at all” to 10 “very helpful	A: scale from 0 “not helpful at all” to 10 “very helpful
Perceived level of psychosocial distress°	Q: “How much distress have you been experiencing in the past week including today?”	Q: “How do you perceive the level of distress of the patient?”
	A: Scale from 0 “no distress” to 10 “extreme distress”	A: scale from 0 “no distress” to 10 “extreme distress”
Content of psychosocial distress°	Q: “What are your greatest burdens?”	Q: “What are the patient’s greatest burdens?”
	A: open answer field	A: open answer field
Trust in oncologist	Q: “How comfortable do you feel talking to the oncologist about personal issues?”	-
	A: scale from 0 “not at all” to 10 “very much”	-

*Q* Question/Item, *A* Answer format. °Questions repeated in patients’ follow-up interview (T2)

### Psychosocial distress measures

Distress Thermometer (DT).

We use the German version of the NCCN Distress Thermometer with Problem List (PL) as the screening tool (T0) for self-reported psychosocial distress, and to identify the causes of expressed distress [6, 28]. The DT is well-validated as a reliable screening tool and has proven itself in clinical practice; it is short and easy to administer [1–5]. The DT contains one item: “Please circle the number [0-10] that best describes how much distress you have been experiencing in the past week including today.” Patients answer on a vertical visual analogue scale from 0 (“no distress”) to 10 (“extreme distress”). We use the cut-off score of 5 or greater, which Mehnert and colleagues suggest indicates a clinically significant level of distress [28]. The PL comprises five problem categories (practical problems, family problems, emotional problems, spiritual/religious concerns, physical problems), and a total of 36 potential causes of expressed distress, each of which can be answered ‘yes’ or ‘no’.

#### Hospital Anxiety and Depression Scale (HADS)

The HADS is a 14-item self-administered questionnaire widely used to detect anxiety and depression in physically ill patients, including cancer patients, and is validated for the German language [29]. The questionnaire has two subscales (anxiety and depression) of seven items each, and a total score for each subscale (values from 0 to 21). Subscale scores between 0 and 7 indicate normal anxiety and depression levels, scores between 8 and 10 indicate borderline levels of anxiety and depression, and scores between 11 and 21 indicate clinical levels of anxiety or depression [30]. The questionnaire is administered to participants at baseline (T1) and follow-up (T2).

#### Fear of Progression Questionnaire (FoP-Q-SF)

The Fear of Progression Questionnaire short form (FoP-Q-SF) is a 12-item self-report questionnaire used to assess the fear of disease progression in physically ill patients [31]. The German version of the FoP-Q-SF is validated in cancer patients and is a reliable instrument, and a total sum score (higher values indicate higher levels of fear of progression) without a standardized cutoff score for clinically relevant level of fear of progression [32, 33]. The questionnaire is administered to participants at baseline (T1) and follow-up (T2).

#### Health-related quality of life (FACT-G7)

The German 7-item version of the Functional Assessment of Cancer Therapy - General (FACT-G) was chosen to assess health-related quality of life in cancer patients at baseline (T1) and follow-up (T2) [34]. The scale comprises three physical well-being items (fatigue, pain, nausea), one emotional well-being item (worry about condition worsening), and three functional well-being items (enjoyment of life, satisfaction with life, sleep). The recall period is the past seven days, and answers range from 0 (“not at all”) to 4 (“very much”) on a 5-point Likert-type scale. The total is the sum of all scores; higher values reflect higher health-related quality of life.

### Social support and coping measures

ENRICHD social support inventory (ESSI).

The ESSI is a reliable and valid 5-item self-report measurement of perceived social support in physically ill patients [35]. We use the German version of the ESSI, which has good psychometric properties [36]. Answers are given on a 5-point Likertscale from 1 (“never”) to 5 (“always”). Scores are summed (range 5–25) and higher scores indicate higher levels of perceived social support. Scores are dichotomized into high and low social support. Low social support is defined as a score of 18 or less, with at least two items that score 3 or less [36]. The questionnaire is administered to participants at baseline (T1) and follow-up (T2).

#### Other support services

Participants are asked if they attend psychosocial support services outside of the University Hospital Basel (assessed at T1 and T2; including psychiatric, psychological, or psycho-oncological support, social service, pastoral care, alternative medicine, complementary medicine).

#### Coping measures

Several questions elicit details on a patient’s subjective perception of how they are coping with cancer, including: perceived threat (“How threatening is the illness to you right now?”, scale from 0 “not threatening at all” to 10 “very threatening”); self-evaluation of coping (“How well are you dealing with your illness at the moment?”, scale from 0 “not good at all” to 10 “very good”); resources for coping (open question: “Who or what has helped you so far in dealing with your illness?”); and, need to talk with someone (“Do you perceive a need to talk with someone about your illness?”, from which patients can select either with friends/family, with a professional person, with both, or with no one).

### Patient-physician communication

Table 1 gives an overview of variables that shape the perception of the first consultation, from the patient and oncologist perspectives.

### Patients’ perception of the conversation with the oncologist

Several questions in the baseline interview address the patient’s perception of the conversation about psychosocial issues and psycho-oncological support with the oncologist during their first consultation (T1; details see Table 1).

### Oncologists’ perception of the conversation with the patient and evaluation of distress screening

Oncologists are asked to evaluate, on a paper-and-pencil questionnaire, their view of the conversation about psychosocial issues and psycho-oncological support options based on the DT in the first consultation (T0; details see Table 1). Reasons for not talking about psychosocial distress with the patient and reasons for not recommending psycho-oncological support are assessed in open answer fields. Oncologists are also asked to assess the usefulness of the DT (“How helpful was the DT in the consultation with the patient?”), rated on a scale from 0 (“not helpful at all”) to 10 (“very helpful”). If the oncologist found the DT helpful, they are asked to specify why, with a choice of six pre-formulated answers (multiple responses possible):

1. “The DT was helpful to assess the patient's distress.”
2. “The DT was helpful to assess the patient’s problems.”
3. “The DT was helpful to initiate the conversation about psychosocial distress.”
4. “The DT was helpful to structure the conversation about psychosocial distress.”
5. “The DT was helpful for referral to psycho-oncological support service.”
6. “The DT was helpful for referral to social care or pastoral care.”

### Open ended questions to assess attitude and reasons for or against uptake

Patients’ attitudes towards psycho-oncological support.

At baseline, attitude towards psycho-oncological support for cancer patients in general is assessed (T1) on a scale from 0 (“not meaningful at all”) to 10 (“very meaningful”). Patients are also asked what expectations and fears they have about psycho-oncological support (open-ended question), if they have ever used psychological support services (yes, no), and how they evaluate their experience, from 0 (“not helpful at all”) to 10 (“very helpful”).

#### Participants’ intention and reasons for (non-) uptake of psycho-oncological support

At baseline (T1), we assess participants’ prospective intention to use psycho-oncological support services (“Do you intend to uptake the in-house psycho-oncological support service in the next months?” answer options: yes, maybe, no), and their reasons (in an open-ended question: “What are the reasons why you do [not/may] intend to use the in-house psycho-oncological support service?”). At follow-up (T2), we use an open-ended question to assess patients’ retrospective reasons for using or refusing psycho-oncological support services in the last four months (“What are the reasons why you did/ did not use the in-house psycho-oncological support service in the last months?”). We analyze the content of responses to open-ended questions (see below).

### Statistical methods

#### Sample size estimation

We estimate recruitment period will last 26 months based, since about 600 new patients attend the oncology outpatient clinics per year. We plan to enroll 700 patients during this period, expect an attendance rate of 20% for the psycho-oncological support service, and a dropout rate of no more than 25%. We estimate that 140 of our study patients will have an outcome, which gives us the power to spend around 9 to 14 degrees of freedom (10–15 events per degree of freedom) in the final regression model and to avoid overfitting the model.

### Statistical analysis

This project allows us to address questions related to the uptake or non-uptake of psycho-oncological services. We are primarily interested in individual patient factors, and physician-related factors that explain and predict uptake of psycho-oncological services. Statistical methods are thus specified separately for each research question. To describe the population characteristics of enrolled patients, we will display the frequency distributions for categorical data and means or medians for continuous data.

The primary outcome and aim of the project are patient and physician factors of psycho-oncological service uptake. The primary outcome of service uptake or non-uptake is defined and ascertained as a binary variable, which we will analyze with logistic regression analysis. To develop explanatory models for the primary outcome, we will first consider expert knowledge to define candidate predictors and potential interactions. We will also explore alternative candidate selection techniques as described by Harrell [37] and Steyerberg [38], and compare the properties of the different models. For continuous predictors (patient age, DT measurement, etc.), we will check the linearity assumption with restricted cubic spline transformations [37, 38]. To deal with potential missing covariate values, we will use multiple imputation and compare the complete case analyses.

Analyses that focus on oncologist perceptions of patients’ distress and their need for psycho-oncological service referral will cluster within physicians. In these situations, we may use robust estimation or random-effects modeling to account for clustering. We may use intra-class correlation to assess variance components between physicians for numeric data and contingency table analyses, or hierarchical modelling to assess paired patient-physician consultation data with binary response variables.

If there was a follow up assessment, the data of that assessment will be entered for analyses. We will use descriptive methods to summarize the frequency (categorical variables) or the distribution of continuous (means [SD] or median [IQR]) baseline variables and the frequency distribution of the dependent variable. We will also run and report comprehensive analyses of missing data and drop out. We will use logistic regression analysis to test the impact of the predictor variables (see, “objectives and research question”) on the outcome use or non-use of psycho-oncologic support.

We will assess univariate associations in logistic regression analysis. We will select relevant predictor variables for multivariable models using univariate pre-selection, based on a liberal *p*-value of *p* < 0.2 [37, 38]. Other than adjusting for age and gender, we will retain significant predictors in the multivariable model based on a type-1 error rate of 5%. For continuous predictors, we will also study non-linear associations using restricted cubic spline transformations with 3–5 knots [37, 38]. Independent variables that arise from patient-physician communication will be “nested within physicians”. We will thus consider using multilevel modeling to account for within physician correlation. If within patient correlation (intra-class correlation) is low (e.g. < 0.05), this will indicate that the variance components between physicians are low. In this situation, regression models for the total patient sample will reveal unbiased SEs. To check agreement between patient and physician perceptions, depending on the nature of the measurement, we will use contingency table analyses or compute the intra-class correlation.

### Analysis of qualitative data

Interviewers will be trained to note key messages of patients’ answers to open-ended questions. Patients’ answers will be recorded in first person. We will use Content Analysis to analyze responses to open-ended questions [39], in MAXQDA 12 (VERBI Software, Germany), a qualitative data analysis software program. A team of trained researchers will discuss the responses to guarantee high quality content analysis. Cohen’s kappa statistics (κ) will be used to assess inter-rater reliability between independent raters.

## Discussion

Many patients with high distress levels do not want psycho-oncological support [40]. This prospective observational study will help us identify predictors and barriers to psycho-oncological support service uptake along the distress screening pathway. We will learn what patients and oncologists think of their communication about psychosocial distress, based on results from a distress screening tool.

We believe this is the first study to consider factors along the distress screening and referral pathway to map the decision process of patients. We 1) combine standard assessment with qualitative data collection, 2) embrace patient and oncologist perspectives, and, 3) focus on communication-related aspects of the distress screening procedure.

Clinical practice and research show that severity of distress is not the only deciding factor in whether a patient accepts a referral to psychosocial services. The presence of clinically relevant levels of psychological distress does not necessarily translate into a patient’s desire for referral for treatment, but patients with negative screens may ask for psycho-oncological services, e.g. [17–19].

### Patient characteristics linked to support service uptake

Research has linked older age [15, 18, 20] and lower education [15, 21, 23, 24] to less service use, even when older and less-educated patients have higher levels of distress, pain, and fatigue. Some studies report that women [18, 21, 22] are more likely to be referred to psycho-social services. But Waller [15] found that very fatigued women were less likely to access services than very fatigued men. Other contextual factors, including treatment modalities, were associated in different ways with patients’ desire for psychological support. Evidence on additional patient characteristics and clinical aspects associated with acceptance of psycho-oncological support other than distress is rare, and participants and study designs were heterogeneous.

### Patient-physician conversation based on results from a distress screening tool

Communication about psychosocial issues is delicate. There is evidence that clinicians do not systematically inquire into the emotional problems of patients, and many clinicians prefer patients to bring up a problem. On the other hand, patients are reluctant to disclose problems [41]. They may have trouble sharing emotional difficulties, and some do not want to address distress and all [42]. In distress screening, we do not know how patients perceive the following conversation about psychosocial issues.

Effective patient-clinician communication encourages patients to openly express psychosocial needs, and to receive and understand information. The perspectives of patients and clinicians must be aligned in a patient-centered communication process designed to overcome barriers to effective communication [43]. Bultz, et al., [26] emphasize that interacting with the patient is the essential element of an effective screening procedure. Despite this, no screening tool offers detailed recommendations to guide physician interaction and communication with the patient. Discussing a patient’s distress score on a screening tool opens an opportunity for physician and patient to effectively communicate about psychosocial issues and psychosocial health needs. Mitchell [44] reported that a screening tool like the Distress Thermometer positively influenced communications about psycho-social issues and distress; clinicians believed the screening program improved communication in more than 50% of assessments. Ours will be the first study to give attention to patient-physician communication stimulated by a distress screening instrument, and to ask how both patient and physician perceive the process.

### Patient reasons for or against support service uptake

In their review, Dilworth and colleagues [27] describe the primary patient-reported reason for refusing support services as, “no subjective need for psychosocial services” (38.7% of pts). This broad reason could include, e.g., a preference for self-managing symptoms, not feeling distressed enough, the belief that help would be ineffective, and receiving sufficient support from family and friends. The second most important barrier in the Dilworth review is context-related. Patients reported they lacked information about the availability of psychological support services (19.0% of pts).

Studies that investigate the reasons patients choose or refuse psycho-oncological assistance rarely include qualitative aspects. A recent study [40] reported that, even in patients with high distress scores, a patient’s preference for self-help and their belief that their distress is not severe enough are common barriers. Mosher [45] had similar results, and also identified inadequate knowledge of services as a patient-reported barrier. A qualitative study found that both a patient’s desire for normalcy and their lack of information about the potential benefits of psycho-oncological treatment could lead patients to refuse psycho-oncological support. The subjective norms and information deficits of physicians also influenced the choice of patients to use psycho-oncological support services [46].

### Limitations

The mono-center setting is both an asset and a liability. Conducted at one University Oncology Outpatient Clinic, our observational study is embedded in a clinic culture that takes a well-accepted interdisciplinary approach, including systematic integration of the psycho-oncological support service team. A study coordinator on the oncology team can closely monitor procedure. The single setting, however, may limit generalizability of our results.

Funding limits permits us only four months of follow-up, so we will not be able to draw long-term conclusions about uptake of psycho-oncological support at later stages of treatment, or in transition phases of illness.

The interviews we conduct may affect participant uptake of support services. This non-interventional observational study will interview participants twice, and ask them why they would or would not want to use psycho-oncological support services. Being asked these questions may motivate and interest participants in using support services. We cannot exclude this effect but, in the follow-up interview, we will ask the participant if this was one reason they used services.

### Conclusions

To raise the quality of psychosocial cancer care, we need to move beyond simple diagnosis and consider the screening process as whole, from a health care delivery perspective. Better understanding the perspectives and potential difficulties in the communication process will help us craft recommendations to improve communication guidelines for distress screening. If we better understand determinants and barriers along the distress screening pathway, we may be able to increase access for underserved groups of distressed cancer patients. We hope to identify routine practices that can lower or eliminate barriers to adequate health care, and better meet patient needs, so we can deploy resources in psychosocial cancer care more efficiently and manage patients better.

## Abbreviations

DT: Distress thermometer
ECOG Scale: Patient performance status according to Eastern Cooperative Oncology Group Scale
EKNZ: Ethikkommission Nordwest- und Zentralschweiz
ESSI: Social support inventory
FACT-G7: Health-related quality of life
FoP-Q-SF: Fear of progression questionnaire
HADS: Hospital anxiety and depression scale
PL: Problem list
